# Evaluation of Computationally Designed Peptides against TWEAK, a Cytokine of the Tumour Necrosis Factor Ligand Family

**DOI:** 10.3390/ijms22031066

**Published:** 2021-01-21

**Authors:** Miriam Badia-Villanueva, Sira Defaus, Ruben Foj, David Andreu, Baldo Oliva, Angels Sierra, Narcis Fernandez-Fuentes

**Affiliations:** 1 Laboratory of Molecular and Translational Oncology, Centre de Recerca Biomèdica CELLEX, Institut d’Investigacions Biomèdiques August Pi i Sunyer (IDIBAPS), 08036 Barcelona, Spain; miribv84@hotmail.com (M.B.-V.); rfoj@leti.com (R.F.); 2 Proteomics and Protein Chemistry Group, Department of Experimental and Health Science, Pompeu Fabra University, Barcelona, Biomedical Research Park, 08003 Barcelona, Spain; sira.defaus@upf.edu (S.D.); david.andreu@upf.edu (D.A.); 3 Structural Bioinformatics Lab (GRIB-IMIM), Department of Experimental and Health Science, Pompeu Fabra University, Biomedical Research Park, 08003 Barcelona, Spain; baldo.oliva@upf.edu; 4 Laboratory of Oncological Neurosurgery, Hospital Clinic de Barcelona—IDIBAPS, 08036 Barcelona, Spain; 5 Department of Biosciences, U Science Tech, Universitat de Vic-Universitat Central de Catalunya, Vic 08500 Catalonia, Spain

**Keywords:** TWEAK, protein-protein interactions, peptide design, orthosteric peptides, expression microarrays, surface plasmon resonance

## Abstract

The tumour necrosis factor-like weak inducer of apoptosis (TWEAK) is a member of the tumour necrosis factor ligand family and has been shown to be overexpressed in tumoral cells together with the fibroblast growth factor–inducible 14 (Fn14) receptor. TWEAK-Fn14 interaction triggers a set of intracellular pathways responsible for tumour cell invasion and migration, as well as proliferation and angiogenesis. Hence, modulation of the TWEAK-Fn14 interaction is an important therapeutic goal. The targeting of protein-protein interactions by external agents, e.g., drugs, remains a substantial challenge. Given their intrinsic features, as well as recent advances that improve their pharmacological profiles, peptides have arisen as promising agents in this regard. Here, we report, by in silico structural design validated by cell-based and in vitro assays, the discovery of four peptides able to target TWEAK. Our results show that, when added to TWEAK-dependent cellular cultures, peptides cause a down-regulation of genes that are part of TWEAK-Fn14 signalling pathway. The direct, physical interaction between the peptides and TWEAK was further elucidated in an in vitro assay which confirmed that the bioactivity shown in cell-based assays was due to the targeting of TWEAK. The results presented here are framed within early pre-clinical drug development and therefore these peptide hits represent a starting point for the development of novel therapeutic agents. Our approach exemplifies the powerful combination of in silico and experimental efforts to quickly identify peptides with desirable traits.

## 1. Introduction

Protein–protein interactions (PPIs) are essential in the shaping of normal and pathological behaviour in cells. Regarding the latter, and although initially deemed undruggable, PPIs are now recognized as important therapeutic targets, hence the development of PPI-modulating drugs is increasing its relevance in pharmaceutical research (reviewed in [1,2]). At the same time, there is a growing perception that the chemistry in classical, small molecular weight (MW) drug discovery programs is not ideally suited for targeting PPIs, as the PPI surface area far exceeds that of conventional organic molecules [3]. Against this background, peptides are emerging as promising candidates for PPI modulation [4]. Small-to-medium size peptides or peptidomimetics are 1–2 orders of magnitude larger than typical low MW drugs, and hence can ideally spanning a much larger surface area. At present, over 60 peptide therapeutics are on the market, and several hundred are under active preclinical and clinical development [5].

The tumour necrosis factor-like weak inducer of apoptosis (TWEAK) is a typical member of the tumour necrosis factor (TNF) ligand family, constitutively expressed in monocytes and some tumour cell types and initially shown to induce apoptosis of malignant cells (reviewed in [6]). The only TWEAK receptor reported so far is fibroblast growth factor–inducible 14 (Fn14), a type I transmembrane protein [7,8]. Fn14 contains the extracellular domain that binds to TWEAK as well as the cytoplasmic tail region essential for signal transduction [9]. Both TWEAK and Fn14 show low expression in normal tissues but are highly expressed in many carcinomas and metastases, associated with a worse clinical outcome [10]. In fact, the so called TWEAK-Fn14 axis is overexpressed in solid tumours [6,11], glioma [12], lung cancer [13,14], melanoma [6], and squamous cell carcinomas [15]. Additionally, Fn14 is highly expressed in the metastases of breast, colorectal, melanoma, non-small cell lung, and prostate cancer [6] and reported to play a role in muscle wasting and cachexia [16,17] as well as in chronic human inflammatory pathologies such as neurodegenerative, autoimmune, or malignant diseases [18]. In this context, specific therapies targeting the TWEAK-Fn14 axis are actively sought as proved by efforts in the field, e.g., [6,11,17,19,20,21]. One of our research priorities is to target the TWEAK-Fn14 axis as potential avenue to prevent brain metastasis, since TWEAK has been found overexpressed in the invasive front of these metastasis lesions from breast cancer patients [22].

Here we report the discovery of four peptides able to target TWEAK and thus modulate the interaction between TWEAK and Fn14. Starting from the 3D structure of TWEAK bound to a neutralizing antibody, a large set of peptides was designed using PiPreD, our structure-based computational approach [23]. A set of 50 designed peptides were selected, based on predicted binding energies and mimicry of important native interface residues (hot spots), and then moved forward to experimental validation. The validation process involved a first stage of cell-based assays followed by confirmation of positive hits by in vitro binding analysis using surface plasmon resonance (SPR). To simplify the screening, the 50 designed peptides were pooled in six batches and both TWEAK-dependent brain metastatic (BRV5) and TWEAK-independent parental breast cancer cells (435-P) were treated at regular intervals with peptides in the presence or absence of TWEAK. mRNA was extracted and the level of expression of nine selected genes from TWEAK-Fn14 signalling pathway was assessed using specifically designed microarrays. Pools showing down-regulation of these genes were selected and individual peptides then evaluated separately. The decomposed pools showed that four individual peptides induced down regulation of probe genes. These four peptides, together with two peptides showing no effect on the cell screening, i.e., negative hits, were selected to measure in vitro binding to TWEAK in a biophysical assay using SPR. Binding data from SPR analyses confirmed that the hits found in the cell-based assays were indeed binding to TWEAK. 

## 2. Results

### 2.1. Computational Design of Peptides

The structure of a neutralizing anti-TWEAK antibody (Ab-T) bound to TWEAK (PDB code 4HT1 [24]) was used as starting point for the design of the peptides. The interface between antibody and TWEAK is composed of 25 anchor residues accounting for a ca. 900 Ang^2^ surface (Supplementary Materials Figure S1). PiPreD incorporates native elements of the protein complex(es) of interest in the modelling and designing process of peptides by the use of the so-called anchor residues as described before [23]. 

In the first step of the modelling stage, over 1.4 million peptides were structurally modelled. The longest distance explored during this modelling stage was 32 Ang; distance spanned between anchor residues Q55 (heavy chain) and S67 (light chain). Prior to the design step, peptides displaying serious steric conflicts (i.e., unlikely to be resolved during the computational design phase) or poorly packed against the surface of TWEAK were discarded, narrowing the starting set to 1.1 million. Of those, around 650,000 were predicted to have mostly helical conformation and were also not considered. The final pool was thus reduced to 423,948 peptides, with sizes ranging from four and 19 residues, that were subsequently ranked based on: (i) predicted binding energy; and (ii) interface packing and mimicry of native interface residues predicted as critical for the interaction by PCPRi [25]. A total of 50 peptides were finally selected for synthesis and experimental validation.

### 2.2. Characterization of Brain Metastatic Cell Lines for Cell-Based Screening of Peptides

**Figure 1:**
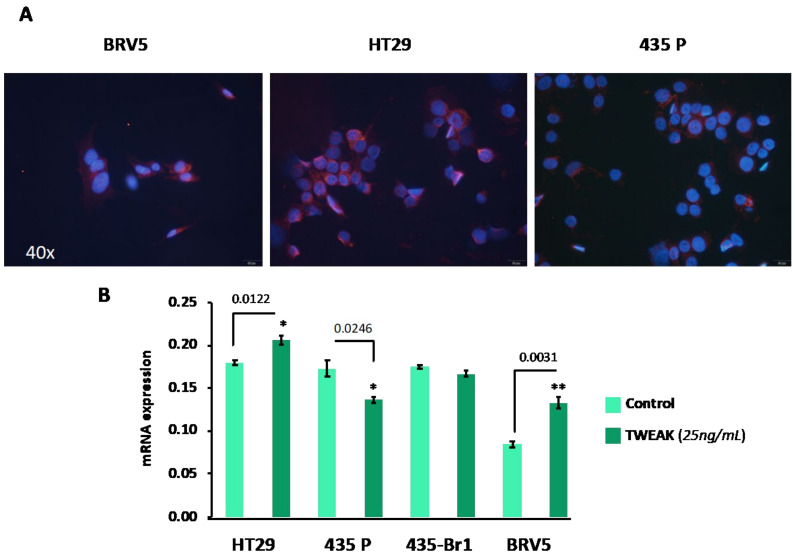
Characterization TWEAK-dependent/not dependent cells. (**A**) Representative images of Fn14 expression (pink) in BRV5, 435-P and HT29 cells labelled by immunofluorescence. The protein has similar expression in BRV5 and the positive control HT29 cells, and less expressed in 435-P. DAPI (blue) was used for nucleus identification. (**B**) RT-PCR analysis to quantify Fn14 gene expression of cells cultured in the presence (dark green) or absence (light green) of TWEAK (25 ng/mL) at 48 h. Up-regulation of Fn14 is observed only in HT29 and BRV5 cells. Bars and error bars represent the average and standard deviation as calculated from three replicates respectively. P values represented as: * - significant (Pvalue: 0.01 to 0.05) and **- very-significant (Pvalue: 0.001 to 0.01).

Fn14 expression is strongly enhanced in human colon carcinoma cells HT29 compared to normal cells and other different transformed tissues. HT29 are human colon carcinoma cells that overexpress Fn14. We used these cells, HT29, as a prototype to evaluate the relative expression of Fn14 in the BRV5 brain metastatic cells; the cell line that was use on the cell-based screening. In addition, we used a well-known model of brain metastasis: 435-Br1 from brain carcinoma including the parental cell line 435-P and the most metastatic variant in brain BRV5. Qualitative immunofluorescent analyses of cell cultures showed Fn14 protein is expressed (Figure 1A) with similar intensity and localization in HT29 and BRV5 cells. In contrast, breast cancer 435-P parental cells, from which BRV5 was derived, had lower staining intensity indicating less Fn14 protein expression. 

Since the transcriptional activation of Fn14 expression can be induced in cancer cells by the TWEAK-Fn14 interaction, either in a paracrine or autocrine manner [9], we investigated whether TWEAK transactivates Fn14 (TNFR2F12A) in BRV5 brain metastatic cells. We primed cells with TWEAK and analysed TNFR2F12A gene expression including in the analysis 435-Br1 cells, the first generation of brain metastatic cells BRV5. Transcriptional expression of Fn14 was significantly activated in BRV5 cells (*p* = 0.0031), similar to control HT29 cells (*p* = 0.0122), suggesting TNFR2F12A dependence from TWEAK levels (Figure 1B and Supplementary Materials Table S2). In contrast, TWEAK decreased TNFR2F12A gene expression in 435-P cells with regard to controls (*p* = 0.0246) and no differences were found in TNFR2F12A gene expression in 435-Br1 cells challenged with this cytokine. These results indicated that expression of TNFR2F12A was independent of TWEAK in 435-P and 453-Br1 cells (i.e., parental and in cells with low metastatic ability); whereas it was induced on TWEAK-dependent cells: HT29 and BRV5 associated to TWEAK-Fn14 axis activation. 

**Figure 2:**
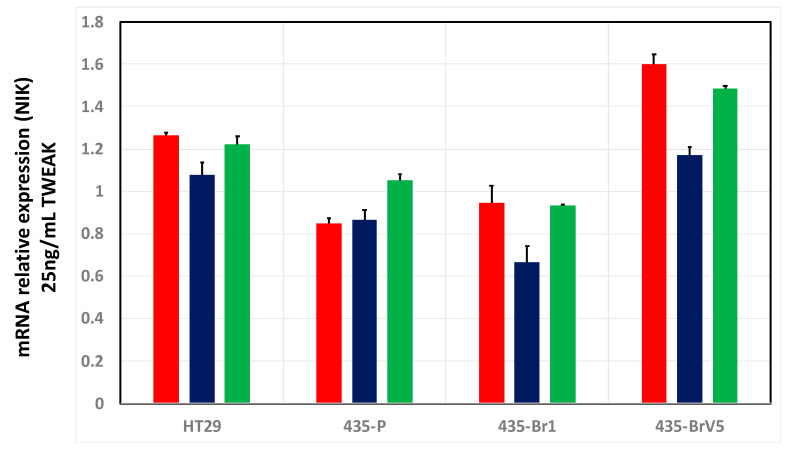
NIK activation in brain metastatic cells. Metastatic cells were primed with TWEAK in a time-course RT-PCR experiment. The bar chart shows activation of NIK (shown as mRNA relative expression) at 6 h (red), 12 h (blue) and 24 h (green). Bars and error bars represent the average and standard deviation as calculated from three replicates respectively.

The final aspect to consider in the characterization of BRV5 cells prior to the screening of peptides was to assess when the signalling pathway mediated by TWEAK-Fn14 will be at its peak of activity. To that end, we monitored the level of expression of the primary kinase NIK (MAP4K4) at different time points. It has been reported that transcriptional activation of Fn14 would be operated mainly by the NF-κB non-canonical pathway, through inducing the primary kinase NIK (MAP4K4) that phosphorylates IKKα [26]. Thus, we assessed the level of expression of NIK from 6 to 24 h after cells were treated with TWEAK in a time-course RT-PCR (Figure 2). These results showed that the highest NIK expression, occurred 6 h after different cell cultures were challenged with TWEAK in both HT29 positive control cells and in the more aggressive brain metastatic BRV5 cells. As expected, TWEAK independent cells, such as 435-P and 435-Br1, showed similar and lower NIK mRNA quantities whereas the expression of NIK increased in TWEAK stimulated BRV5 cells after 6 h of treatment, decreasing at 12 h and partially recovered at 24 h. From these experiments we concluded that TWEAK-Fn14 pathway is mainly activated at 6 h after treating cells, and thus we designed out experiments to check the inhibitory ability of the peptides at this specific time point. 

### 2.3. Selection of Genes for Cell-Based Screening of Peptides

**Figure 3:**
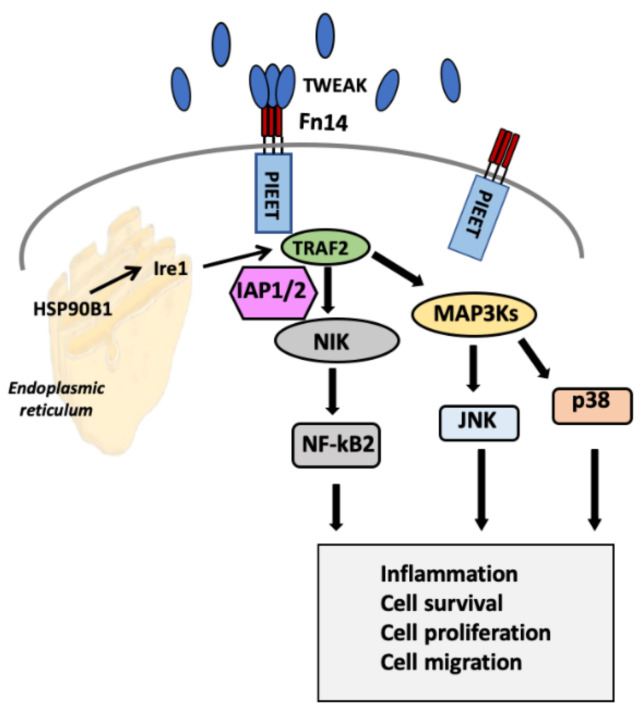
Simplified view of the TWEAK-Fn14 signalling pathway. The selection of the probe genes to be monitored upon treatment with peptides and/or TWEAK was based on the analysis of Reactome [27] and NetSlim [28] databases, and a manual curation of TWEAK-Fn14 signalling pathway [29]. Downstream TWEAK-Fn14 activation complex, recruitment of the TRAF cofactor molecules, through NIK stabilization, engages ERSRP and non-canonical activation of NFkB. MAP3Ks cascades activation lead JNK (c-Jun N-terminal kinase) and engagement of p38 (MAPK14), which results in their translocation to the nucleus, wherein they modulate gene expression.

The selection of the probe genes to be monitored upon treatment with peptides and/or TWEAK was based on the analysis of Reactome (https://reactome.org/content/detail/R-HSA-5676550) [27] and NetSlim (http://www.netpath.org/netslim/tweak_pathway.html) [28] databases, and a manual curation of TWEAK-Fn14 signalling pathway [29]. Figure 3 presents a summarized version of downstream effectors considered for monitoring as well as the different pathways linked. Thus, TWEAK-Fn14 activation in BRV5 brain metastatic cells was monitored looking at the expression of these genes as well as the effect of designed peptides in the level of expression as a proxy of disruption of TWEAK-Fn14 interaction. For a full view of the signalling pathways refer to resources shown above. Overall, we selected 11 genes to investigate: nine on signalling pathways downstream from the TWEAK-Fn14 axis, and two housekeeping genes to normalize the level of expression (more details on material and methods). 

### 2.4. Cell-Based Screening of Pooled and Individual Peptides on Gene Expression Microarrays

To optimize resources and rationalize the analysis, we ran the screening by pooling the 50 peptides into six groups, i.e., Pool 1 to 6, containing 10–12 peptides each and displaying similar MW (Supplementary Materials Table S1). We monitored the activity of pooled peptides on TWEAK-Fn14 signalling pathways following the design described in Material and Methods. BRV5 cells were cultured in the presence or absence of TWEAK and treated with peptides at different time points. 

As a preliminary study, we performed an experiment with 435-P and BRV5 cells to assess the effect on the level of expression on the selected genes in cultures with or without TWEAK (Supplementary Materials Figure S2A). Negative controls without pool 1 peptides were included in the protocol to avoid unspecific biases in the inhibition ratio. To achieve optimal conditions to measure peptide activity, preventing peptide degradation in the culture medium, the effect of peptides was evaluated by priming cultures with peptide every hour (0–5 h), every two hours (0, 2, and 4 h) and every three hours (0 and 3 h). In these experiments, the housekeeping gene NARS2 was used to normalize gene expression. 

As described previously (Figure 2), we expected the activation of TWEAK-Fn14 pathway to be at its peak at 6 h and the most efficient down regulation of genes occurred when peptides were added every hour. Downstream activation of MAP4K4 (NIK), TRAF2, and BIRC2 (IAP2) occurred at 6 h in BRV5 cells but not in TWEAK independent 435-P cells (Supplementary Materials Figure S2B). These exploratory experiments provided the best conditions to check the ability of peptides to affect TWEAK-Fn14 pathway and we adopted this protocol for the rest of the pools, i.e., pool 2 to 6.

**Figure 4:**
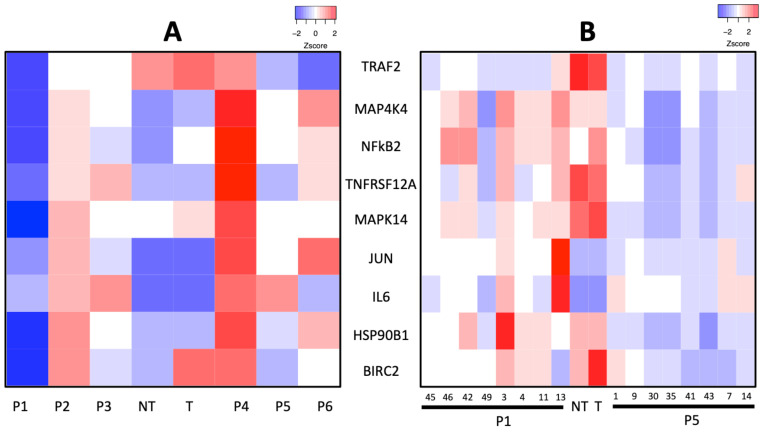
Gene expression of selected genes in TWEAK-Fn14 signalling pathway in response to peptide treatment. (**A**) Heatmap representing the normalized expression of genes (Y axis) in response to the treatment with pooled peptides (Pool 1 to 6; P1, P2, P3, P4, P5 and P6) as well as the response of BRV5 cells not treated (NT) and treated with TWEAK (T) (X axis). (**B**) Heatmap representing the normalized expression of genes (Y axis) in response to the treatment with individual peptides (P1 pool: P45, P48, P42, P49, P3, P4, P11, P13; P5 pool: P1, P9, P30, P35, P41, P43, P7, P14) as well as the response of BRV5 cells not treated (NT) and treated with TWEAK (T) (X axis).

According to this protocol, Figure 4A shows a heatmap of Zscore gene expression values of each reporting gene (normalized by the expression of housekeeping gene NARS2) on BRV5 cells stimulated with TWEAK and treated with a recurring dose of peptides every hour. BRV5 cells stimulated with TWEAK in absence of peptides (T in the heatmap) presented a higher level of expression overall when compared to untreated and unstimulated cells (NT). In particular, it showed higher expression of TRAF2, suggesting the activation of non-canonical NFkB2 pathway also implicated in the transcriptional activation of BIRC2 gene. MAPK14 kinase also shows a slightly higher level of expression as well as NFκB2 gene. 

In pool 1 and 5 (labelled P1 and P5 in the Figure 4A respectively), we observed a downregulation of genes along the TWEAK-Fn14 signalling pathway with all (Pool 1) and 8 out of 9 (Pool 5) reporting genes presenting low expression compared to T band. Pool 3 also seems to have an effect on several genes albeit lower than Pool 1 and 5. Also interesting, although out of the scope of this work and for follow up studies, is the effect observed in pool 4 where there is an upregulation across all monitored genes. As mentioned, this is an interesting observation that could be explained by the methodology used to design the peptides, i.e., elements of the native complex are used to design peptides. Hence, it could be the case that peptide(s) in this pool acts as agonists of Fn14 rather than targeting TWEAK.

**Figure 5:**
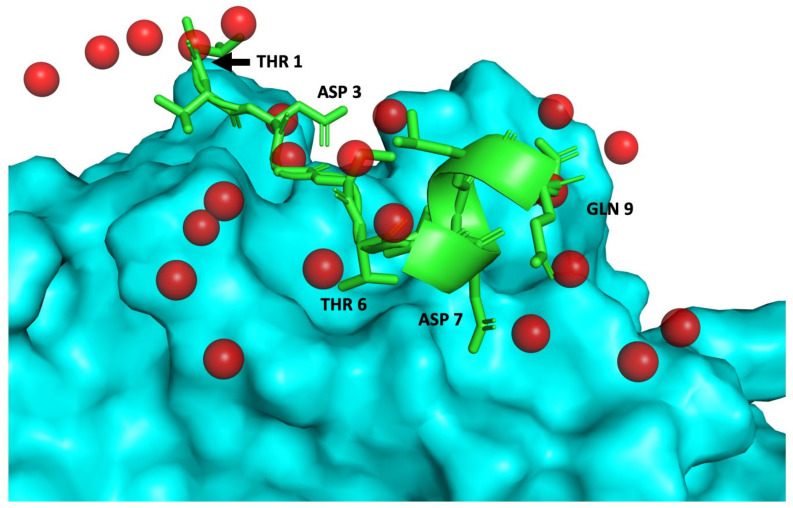
Structural model of PEP43-TWEAK complex. TWEAK and P43 shown in cyan (surface representation) and green (cartoon and stick representation) respectively. Several residues of P43 are shown for reference. Red spheres represent the anchor residues derived from the TWEAK-Antibody structure (PDB code: 4HT1 [24]) used for peptide modelling (see Material and Methods and Supplementary File File1.coord.pdb). The structural rendering of protein and peptides shown in this figure and Figures S4–S6 were done using PyMol (http://pymol.org).

Pool 1 and 5 were therefore deconstructed to assess the effect of individual peptides and their activity on same cell-based screening. Pools 1 and 5 were composed of peptides 45, 46, 42, 3, 49, 11, and 13; and of peptides 1, 9, 30, 35, 41, 43, 7, and 14, respectively. As before, and shown in Figure 4B, the level of expression of probe genes on TWEAK-stimulated (T) BRV5 cells is overall higher than on non-stimulated (NT) one. Among individual peptides, those belonging to Pool 5 show lower expression levels, with peptides 30, 35, and 43 displaying the lowest overall expression across all genes. In the case of Pool 1, peptide 49 induces the lower expression of genes when comparing to the rest of peptides included in the pool. Peptides 3, 4, 11, and to some extent, 13 induced a higher level of expression of the gene suggesting perhaps an activation role as it was found already when the screening was done on the pools of peptides. The structural model of peptides 43, 30, 35 and 49 bound to TWEAK can be seen in Figure 5 and Supplementary Materials Figures S4–S6, respectively. 

The structural models of TWEAK-peptides complexes show that the peptides cover the central region of the interactions between TWEAK—Ab-T. The mimicry of anchor residues is important too, with peptides surrogating 3–7 of the anchor residues. The conformation of the peptides is largely extended with small porting adopting a helical conformation. As described, Ab-T interface with TWEAK overlaps with the interface of Fn14 [24], and therefore these peptides in principle will have an orthosteric effect and compete to prevent the interaction with Fn14.

### 2.5. In Vitro Peptide Screening by Surface Plasmon Resonance

SPR biosensor interaction analysis, a label-free screening approach, was used for real-time monitoring of the kinetics and affinity of peptide hits to TWEAK previously identified by cell-based screening. Peptides from pools 1 and 5 were selected both as positive (49, 30, 35 and 43) and negative hits (1 and 42). For our purposes, TWEAK was chosen as ligand, while peptides were chosen as analytes and passed across ligand-derivatized surface. Nitrilotriacetic acid-coated sensor chips (NTA chip) and a coupling approach [30] that results in the capture of a His6-protein in a non-random orientation by the His6 epitope prior to direct free amine-based covalent coupling was used for histidine-tagged TWEAK immobilization. 

**Figure 6:**
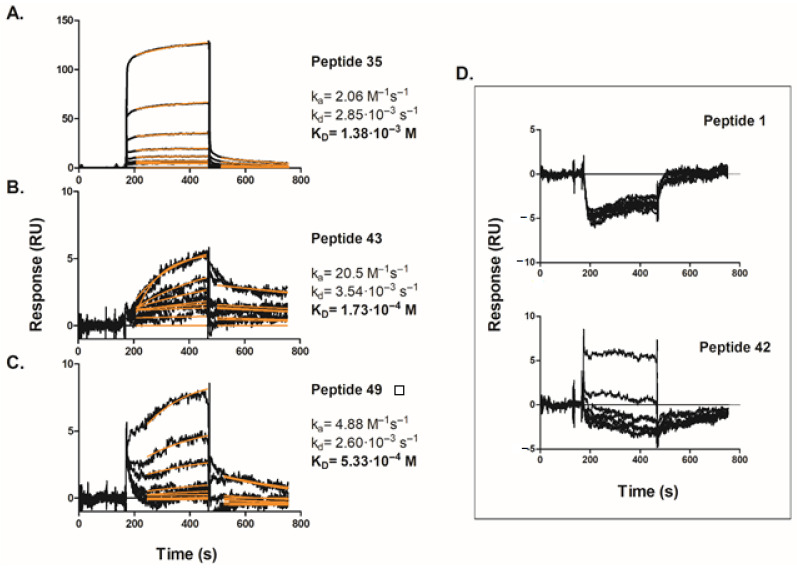
SPR sensorgrams of the selected TWEAK-Fn14 inhibitory peptides binding to immobilized human His6-TWEAK protein: (**A**) peptide 35, (**B**) peptide 43, (**C**) peptide 49 and (**D**) negative control peptides 1 and 42. The experimental data (black lines) were globally fit to a 1:1 binding model (red lines) using BIAevaluation, as described in Materials and Methods, to determine the indicated kinetic rate and affinity constants. Data are representative of at least three independent experiments.

Purified His6-TWEAK protein was successfully immobilized at a moderate density (~7000 RU_immob_) on the NTA sensor chip and the newly created surface was tested for functionality by conducting SPR binding assays using a monoclonal anti-TWEAK antibody (see Supplementary Materials Figure S3) and giving an affinity K_D_ of 75 nM that agrees with the literature values for the cognate TWEAK-Fn14 pair [31]. After chip validation, peptides selected on our cell-based microarray screening were tested. Figure 6 shows the set of binding sensorgrams obtained for various concentrations, ranging from 6.25 µM to 800 µM, of peptides 35, 43, and 49, binding to His6-TWEAK covalently immobilized on the sensor chip surface. Unfortunately, in the case of peptide 30 it was impossible to carry out the experiment, as it precipitated at all concentrations tested. 

For each titration, a zero-analyte surface as a control was subtracted from test sensorgram as well as non-specific binding to the blank flow cell without protein; and the chip surface was regenerated in order to remove any remaining analyte. The black curves show the experimental data, while the overlaid red lines represent a global fit of the entire data set to a 1:1 Langmurian interaction model. The fitting process provided both kinetic association and dissociation rate constants (K_a_ and K_d_), as well as equilibrium dissociation constant (K_D_) values indicated in Figure 6. Good fitting of experimental data to the calculated curves has been observed, suggesting the correctness of the used model. Furthermore, as expected for interactions involving low molecular weight peptides, the dissociation constants obtained were in the micromolar range with the peptide 43 (Figure 6B) presenting slightly higher affinity for TWEAK (i.e., K_D_ of 170 µM). 

In addition, peptides 1 and 42 were chosen and tested as negative controls given its lack of effect against the probe genes. As shown in Figure 6D, both peptides showed negligible binding affinity for immobilized TWEAK; global curve fit was not attempted in this case. These observations provide a convincing evidence that our selected peptides are able to bind TWEAK protein with reasonable affinities values in the 100–500 µM range and further support our genuine peptide hits, as we have observed good data correlation between the in vitro biophysical and cell-based methods for all the tested peptides.

## 3. Discussion

The role of TWEAK-Fn14 has attracted considerable attention within recent years as it has been associated to different cancers and metastasis as well as other conditions (see Introduction). Based on these evidences, efforts are currently directed to target TWEAK using both small molecules [19] and antibodies (reviewed in [6]). Among the latter, it is worth mentioning a humanized antibody anti-TWEAK receptor at different stages on pre-clinical and clinical trials [6,11]. Thus, any improvement in therapies targeting this axis can offer new perspectives in the treatment of several disorders. In this work, we contribute to these efforts with the discovery of novel peptides to target TWEAK protein and thus modulate its interaction with Fn14 receptor. To the best of our knowledge, de novo peptides have been neither used nor described to target the TWEAK protein.

Peptide therapeutics have kept pace with scientific innovation by expanding into new indications and molecular targets and are a natural starting point for drug discovery, as exemplified by the increasing list of peptide drugs in the therapeutic market [5]. The peptides presented here started from the 3D structure of TWEAK bound to a neutralizing antibody, and from this, a large set of peptides was designed using PiPreD, a structure-based computational approach [23]. The selection of 50 peptides based on predicted binding energy and mimicry of native important interface residues (hot spots) yielded four hits as confirmed by two different and orthogonal experimental methods. Thus, our computational strategy was able to narrow down a very large pool of potential peptides sequences (in the order of hundreds of thousands) to a manageable number of them that could be processed on a low throughput manner, hence optimizing the most costly and longer experimental validation. To further optimize our resources, the pooling of peptides in the cell-based assays appeared to be also a valid approach, where instead of starting with individual peptides but pools allowed a more cost-effective and quicker screening. Once the desirable biological effect was observed on the given pool, i.e., downregulation of probe genes, then these peptides were both re-assessed by cell-based assays and further confirmed in vitro.

Looking forward, the results presented here are the first step on a long journey toward new therapeutic avenues. We focused on a breast metastasis where Fn14 is a brain metastasis biomarker predictor since the likelihood to develop brain metastasis in Fn14-positive luminal breast carcinomas increased 36.70-fold [10]. Moreover, Fn14 in Src-regulated cell migration/invasion and activation of NF-κB signaling are implicated in the aggressiveness of non-small cell lung cancer cells [13,32], the source of most brain metastasis cancer. Based on these evidences, many efforts are been currently directed at evaluating whether elevated Fn14-driven activity is a potential therapeutic target to prevent metastasis progression. As mentioned, TWEAK- or Fn14-targeting agents are already been used to inhibit the progression of tumors and have achieved some success in clinical and pre-clinical trials including a humanized antibody anti-TWEAK receptor is in phase 1 safety study in patients with solid tumors [11] and TWEAK and Fn14-targeting agents have already been tested in pre-clinical trials to treat cancer showing encouraging outcomes [6].

## 4. Materials and Methods 

### 4.1. Computational Design, Ranking and Selection of Peptides Mimicking the Interaction of a Neutralizing Antibody with TWEAK

Peptides were modelled using PiPreD [23] using the structure of TWEAK-TWEAK antibody (PDB code 4HT1 [24]). The parameters used in the modelling stage were as follows: (i) a maximum distance between Cα-Cα iMotifs-anchors of 0.5 Ang, and (ii) a root mean square deviation value smaller than 1.0 Ang upon structural superposition iMotifs-anchors. The design of peptides was done using the FIXBB protocol in Rosetta adapted to peptides as previously described [33]. A schematic depiction of the modelling process is shown in Figure S1.

From the original pool of designed peptides, those presenting mostly helical conformation were discarded. From the remaining ones, i.e., peptides with linear conformation, a two-step scoring approach was applied. First, peptides were ranked using the Rosetta-predicted binding energy [34]. Subsequently, they were prioritized on two criteria: (i) mimicry of important interface residues as predicted by our PCRPi method [25]), i.e., peptides including important elements of the native complex were prioritized over those showing comparable predicted binding energy; and (ii) peptides with a higher interface packing. A final selection of 50 peptides was taken forward for experimental validation (Supplementary Materials Table S1).

### 4.2. Peptide Synthesis and Characterization

Peptides were synthesized as previously reported [35]. The synthesis was done in C-terminal carboxamide form by Fmoc solid-phase peptide synthesis on a H-Rink Amide-ChemMatrix resin of 0.50 mmol/g substitution (PCAS BioMatrix, Quebec, Canada) at a 0.05 mmol scale in a Prelude automated synthesizer (Protein Technologies, Tucson, AZ). Side chains of trifunctional residues were protected with TFA-labile *t*-butyl (Asp, Glu, Ser, Thr), trityl (Asn, Gln, His), Boc (Lys), and 2,2,4,6,7-pentamethyldihydrobenzofuran-5-sulfonyl (Arg) groups. Couplings were systematically performed with a 5-fold excess of Fmoc-amino acid in the presence of N,N,N’,N’-tetramethyluronium hexafluorophosphate (5 eq) and N,N-diisopropylethylamine (10 eq) with DMF as solvent. Fmoc removal was done with piperidine/DMF (20:80 *v*/*v*) followed by DMF washes. Cleavage and deprotection of the peptide resins was done with TFA-water-triisopropylsilane (95:2.5:2.5, *v*/*v*/*v*, 90 min, r.t.). Peptides were isolated by precipitation with cold diethyl ether and centrifugation, then solubilized in water and lyophilized. 

As previously reported [35], the synthetic crude products were analysed by analytical RP-HPLC and LC-MS and purified by preparative RP-HPLC. Analytical RP-HPLC was performed on a LC-20AD instrument (Shimadzu, Kyoto, Japan) fitted with a Luna C18 column (4.6 mm × 50 mm, 3 μm; Phenomenex, Torrance, CA, USA) using linear gradients of solvent B (0.036% TFA in ACN) into A (0.045% TFA in H_2_O) over 15 min, at 1 mL/min flow rate and with UV detection at 220 nm. Preparative RP-HPLC was performed on a LC-8 instrument (Shimadzu) fitted with a Luna C18 column (21.2 mm × 250 mm, 10 μm; Phenomenex), using linear gradients of solvent D (0.1% TFA in ACN) into C (0.1% TFA in H_2_O) over 30 min, with a flow rate of 25 mL/min. MS analysis was performed on a LC-MS 2010EV instrument (Shimadzu) fitted with an XBridge C18 column (4.6 mm × 150 mm, 3.5 μm, Waters, Cerdanyola del Vallès, Spain), eluting with linear gradients of F (0.08% formic acid (FA) in ACN) into E (0.1% FA in H_2_O) over 15 min at 1 mL/min flow rate. Fractions of >95% HPLC purity and with the expected mass by LC-MS were pooled and lyophilized. 

### 4.3. Cell Lines

Three different cell lines were used in this study: BRV5, MDA-MB 435 (435-P), and HT29. The BRV5 is a highly metastatic brain cell line, obtained in one of our laboratories by primary culture from a brain of a nude mouse inoculated with human 435-Br1 brain metastatic cells, as previously described and used as a model to test the peptides in cell cultures [36]. This cell line represents a good model to study the inhibition of TWEAK as it is overexpressed in brain metastases [22] and Fn14 gene expression is low in normal brain tissue but up-regulated in advanced brain cancers, with maximal levels in the invading cells within normal brain tissue [37]. The triple negative 435-P cell line [38] is a useful human breast cancer model that expresses both epithelial and melanocytic markers [39], and was included as the parental cell from which 435-Br1 metastatic variant was initially originated (both cell lines kindly provided by Dr Fabra at IDIBELL Institute, Hospitalet de Llobregat, Spain). Finally, HT29 human colon carcinoma cells obtained from the European Type Culture Collection (ECACC 91072201) were used as a positive control of TWEAK dependent Fn14 over-expressing cells to evaluate TWEAK-Fn14 axis of brain metastatic variants. 

### 4.4. Cell Culture Conditions and Treatments for Screening Peptide Activities

Appropriate cell culture conditions were established as the controls needed in the expression of genes with and without peptides and with and without TWEAK activation, both as pools and individually (see Supplementary Materials Figure S2). The protocol started with the seeding of 125,000 cells/well in 500 µL of culture media supplemented with 10% fetal bovine serum (CM+FBS10%) in 24-well dishes for 24 h before removing FBS (CM-FBS). After overnight starvation in CM-FBS culture, different peptide treatment conditions (at final concentration of 450 µM) were established in the presence or absence of TWEAK (25 ng/mL final concentration) (Peprotech EC, Ltd., London, UK). Some cells were treated with peptides at a single, unique, initial dose (time 0) while other were treated with repeated doses to account for the effect cell-culture degradation. These include a repeated treatment every hour (i.e., time 0, 1, 3, 4, 5), every two hours (time 0, 2, and 4) and every three hours (time 0 and 3)—see Supplementary Materials Figure S2 for diagrammatic depiction. At time 6 (after six hours) cells were lysed and RNA extracted for transcriptomic analyses (see next). Cell were culture for six hours as this was the time needed to achieve the highest upregulation of the TWEAK-Fn14 axis upon TWEAK treatment as shown in previous time-course RT-PCR experiments (see Figure 2).

### 4.5. Transcriptomic Analysis

RNA from cells was extracted 6 h after treatments by direct lysis on the well. The RNeasy Micro kit (Qiagen, Hilden, Germany) was used to isolate total RNA from cell lysate following manufacturer’s protocols including an additional step of digestion with DNase I (Qiagen, Hilden, Germany). Total RNA concentration was measured in a NanoDrop 1000 spectrophotometer (Thermo Scientific, Waltham, MA, USA). The RNA extracted from treated and untreated cells (see above) was used as a proxy for gene expression. 

The quantitative RT-PCR was performed using Fluidigm Dynamic Array FlexSix array with the specific primers for gene expression (Delta Genes Assays, Wet lab tested on Dynamic Array IFC) designed to RefSeq for detection by pPCR EvaGreen assay (Dynamic Array FlexSix, Fluidigm Corporation). Each Flex Six IFC has a total of six independent partitions. Each partition has a 12 × 12 format (12 assay inlets and 12 sample inlets) and will be run independently as a separate experimental run. The dynamic array was loaded with the sample and assay mixtures via the appropriate inlets using an IFC controller and the chip placed in the BioMark Instrument according to the protocol GE Flex Six PCR+Melt v1. Data was analysed with Real-Time PCR Analysis Software in the BIOMARK instrument (Fluidigm Corporation) to obtain Ct and delta Ct values. 

The level of expression of 11 genes were investigated; 9 on the signalling pathways downstream from the TWEAK-Fn14 axis, and two housekeeping genes. The downstream genes were: TNF receptor-associated factor 2 (TRAF2), IAPS (BIRC2), NIK (MAP4K4), kappa light polypeptide gene enhancer in B-cells 2 (NFkB2), P38 (MAPK14), interleukin 6 (IL-6), GRP94 (HSP90B1), Fn14 (TNFR2F12A) and JUN proto-oncogene (JNK). The two housekeeping genes were the asparaginyl-tRNA synthetase 2 (NARS2) and mixed-lineage leukaemia AF10 (MLLT10) gene. Data was analysed using the Dynamic Array FlexSix (Fluidigm Sciences, Inc., South San Francisco, CA, USA) using the NARS2 gene to normalize all values and quantify gene expression. Expression data are available in Supplementary File File5.xlsx and Supplementary Materials Tables S3 and S4.

### 4.6. Surface Plasmon Resonance (SPR) Analysis

Binding assays were performed at 25 °C in a Biacore 3000 instrument (GE Healthcare, Uppsala, Sweden) using nitrilotriacetic (NTA) sensor chips (GE Healthcare) and HBS-EP (0.01 M HEPES pH 7.4, 0.15 M NaCl, 3 mM EDTA, 0.005 % *v*/*v* Surfactant P20) as running buffer. Histidine-tagged human TWEAK (His6-TWEAK) protein (R&D Systems, Minneapolis, MN, USA) was covalently immobilized on the sensor surface using a capture coupling method [30]. Briefly, the NTA surface was first stabilized by injection of regeneration buffer (20 μL EDTA-containing running buffer at 20 μL/min) followed by Ni^2+^ binding to the surface (40 μL of 500 μM NiCl_2_ in running buffer at 20 μL/min). The carboxymethyl dextran surface was then activated for primary amine coupling by injecting coupling solution (30 μL of a 1:1 N-hydroxysuccinimide (NHS)/1-ethyl-3-[3-(dimethylamino)-propyl]carbodiimide hydrochloride (EDC) mixture at 5 μL/min). His6-TWEAK protein in pH 7.4 running buffer was then injected over the surface (60 μL of 10 μg/mL His6-TWEAK at 5 μL/min). To block uncoupled primary amines on the sensor surface, ethanolamine was passed over the surface (35 μL 1M ethanolamine at 5 μL/min). Ligand surfaces were finally conditioned with a 20 µL injection of regeneration solution containing 350 mM EDTA at 20 μL/min. Immobilization levels ranged from 1500 to 7000 resonance units (RU_immob_) to test different ligand densities. A blank flow cell on the chip was created by simple injection of 20 μL of regeneration buffer injected over the sensor surface. 

Binding experiments using a monoclonal anti-TWEAK antibody (Abcam ref. 199405, Cambridge, UK) were run to ascertain adequate functionalization of the immobilized surface. Selected synthetic TWEAK-Fn14 inhibitory peptides at two-fold serial dilutions from 800 µM to 0 µM in running buffer were flown over the chip for 5 min at 35 µL/min. The dissociation phase was monitored for 10 min at the same flow rate and followed by a regeneration step with 10 µL of 18 mM NaOH. Specific binding responses for each channel were obtained by double referencing; subtracting non-specific binding to the blank flow cell plus an internal reference standard, namely buffer injection response. Each sample was run in triplicate. The experimental data were fitted to a 1:1 Langmuir binding model by the BIAevaluation software version 4.1 (GE Healthcare, Uppsala, Sweden).

## 5. Conclusions

The interaction of cytokine TWEAK with receptor Fn14 plays an important role in the activation of downstream signalling pathways involved in cell growth and differentiation. It has been shown that this interaction is also important in a number of diseases including cancer and metastases. In this work, we present the screening and validation of computationally designed peptides to modulate the interaction between TWEAK and Fn14. Specifically, we describe four synthetic peptides able to bind to TWEAK. The experimental data show that these peptides can trigger intracellular responses in cells that are dependent on TWEAK for their growth, while they do not induce any phenotypical effect on those which are TWEAK-independent. Gene expression of genes in TWEAK-Fn14 pathway is largely inhibited when cells are treated with the peptides. These peptides have been further assessed in an in vitro assay confirming that TWEAK was indeed the target. Altogether, our findings represent a first account of small peptides able to target the extracellular TWEAK-Fn14 interaction and trigger an intracellular response, as shown on the decrease of expression of downstream effectors. We envision that follow up studies will see these peptides, or derivatives thereof, being used to downregulate TWEAK-Fn14 pathways. Obviously, additional pre-clinical research efforts are still needed in that direction, with our results within this context being just an initial steppingstone for further therapeutic developments. Finally, and although beyond the scope of this work but perhaps warranting further research, we have also found a set of peptides which actually cause the opposite effect, i.e., strong activation of the gene reporters along TWEAK-Fn14 associated pathways, which might be explained by an agonist effect on Fn14.

## Data Availability

The data presented in this study is included in the article and its Supplementary Material. Requests can be also made to corresponding authors.

## Supplementary Materials

Supplementary materials can be found at https://www.mdpi.com/1422-0067/22/3/1066/s1.

## Abbreviations

SPR	Surface plasmon resonance
TWEAK	Tumor necrosis factor-like WEAK inducer of apoptosis
Fn14	Fibroblast growth factor-inducible 14
MW	Molecular weight
