# Supplementary material for: Evaluation of Computationally Designed Peptides against TWEAK, a Cytokine of the Tumour Necrosis Factor Ligand Family
Source: Int J Mol Sci. 2021 Jan 21;22(3):1066. doi: 10.3390/ijms22031066 (PMC7866087; doi:10.3390/ijms22031066)
Supplement: Supplementary file 1 [file ijms-22-01066-s001.zip › suppl/Suppl_revision.pdf]

Supplementary material to

Computational design of peptides to target TWEAK, a cytokine of the tumour necrosis factor ligand family.

Badia-Villanueva et al.

Supplementary Figures

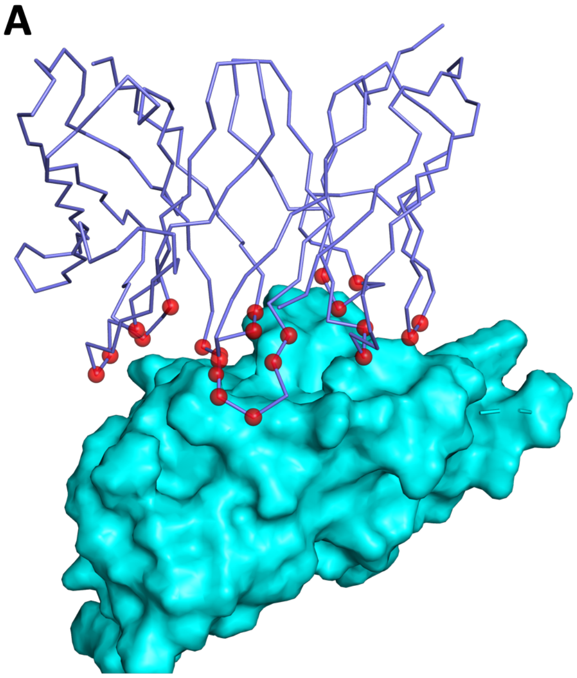

Figure S1 Depiction of the modeling process. A) Structure of the human TWEAK in complex with the Fab fragment of a neutralizing antibody (PDB code: 4HT1[1]); TWEAK and Antibody shown in surface (cyan) and ribbon (light blue) respectively. The C-alpha of the interface residues belonging to the Antibody used to model peptides (*anchor residues*) are shown as red spheres. **B)** Close-up of the interface TWEAK-Antibody. **C)** Structural model of TWEAK and few designed peptides; peptides shown as C-alpha traces.

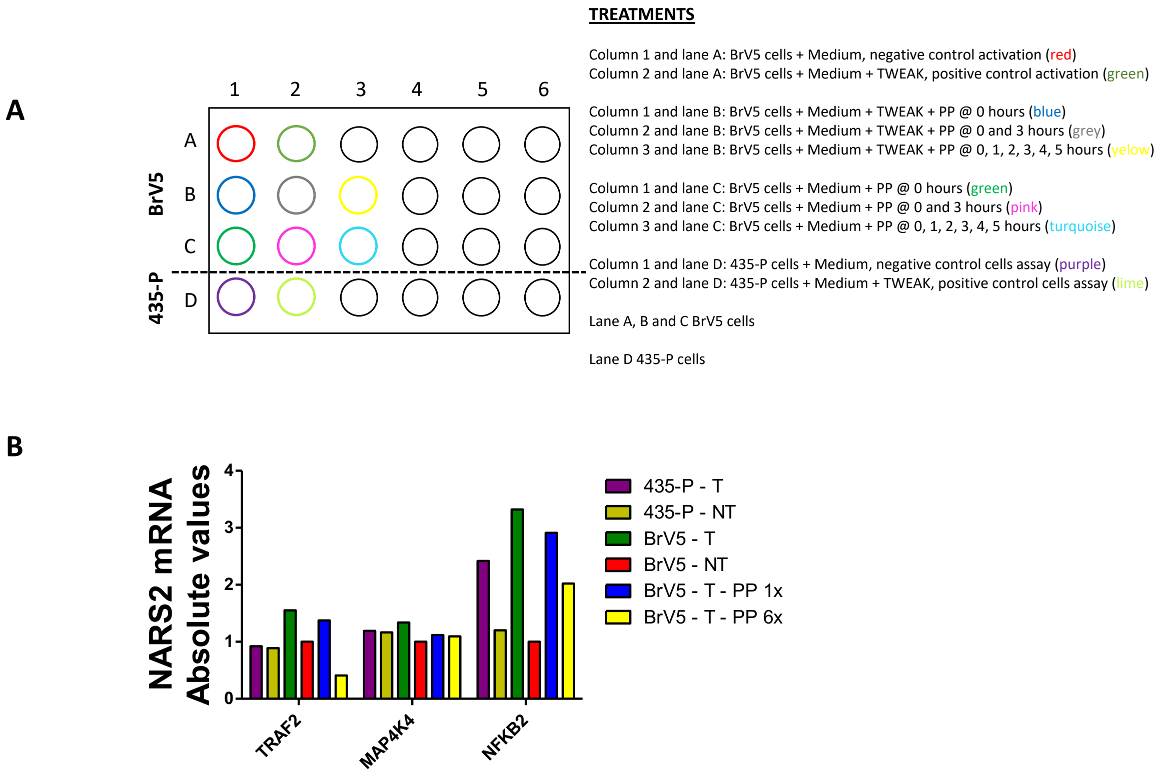

Figure S2. Design of cell array and treatments. A) Schematic representation of the different culture conditions that we performed to analyze gene expression of cells stimulated with TWEAK and the inhibitory ability of peptides to override TWEAK- Fn14 signaling pathway. BrV5 cells were plated in 24 well plates, 125.000 in 500 µl DMEN-F12 with 10% FBS 24 h. After overnight starvation, cultures were treated with or without peptides (450 µM), in the presence or absence of TWEAK (25 ng/mL final concentration), including positive and negative controls to check peptides activity and the inhibitory potential of each peptide concentration according the illustrated scheme. **B)** In addition to the array technique, RNA extracted at 6 h was evaluated with RT-PCR as an alternative technique. The expression of MAP4K4 (NIK), TRAF2 and NFkB2 (NIK) normalized to NARS2 (error bars show s.e.m. of 3 technical replicates) we evaluated in BrV5 cells with (BrV5-T) or without (BrV5-NT) TWEAK. BrV5-T fed each hour, 6 times total (PP x1) and only one time at 0 h (PP x1), are compared. Comparison of gene expression in TWEAK independent cells 435-P with (BrV5-T) or without (BrV5-NT) TWEAK are included in the analyses.

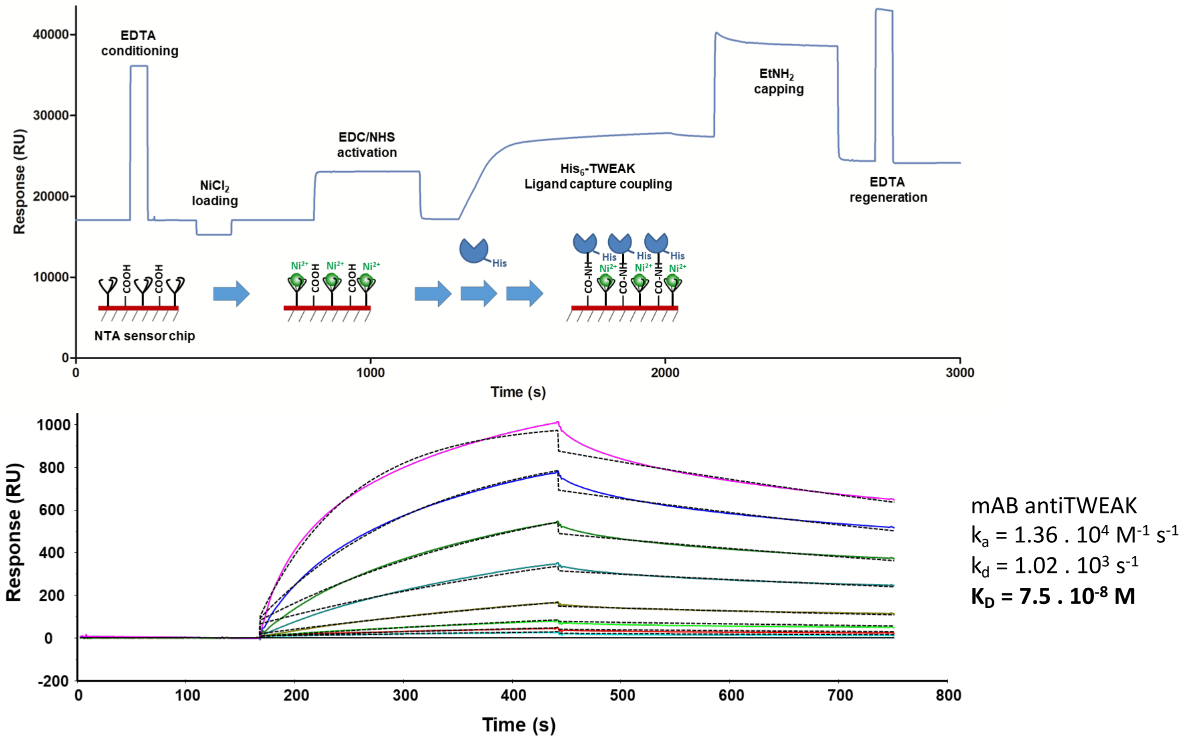

Figure S3. **Immobilization of TWEAK and validation. A)** Sensorgram generated during the immobilization of a single flow cell using an NTA chip and the capture coupling method. **B)** SPR binding sensorgrams of mAb ANTI-TWEAK to immobilized human His6-TWEAK. Experimental data (coloured lines) were globally fit to a 1:1 binding model (dotted black lines) using BIAevaluation to determine the indicated kinetic rate and affinity constants.

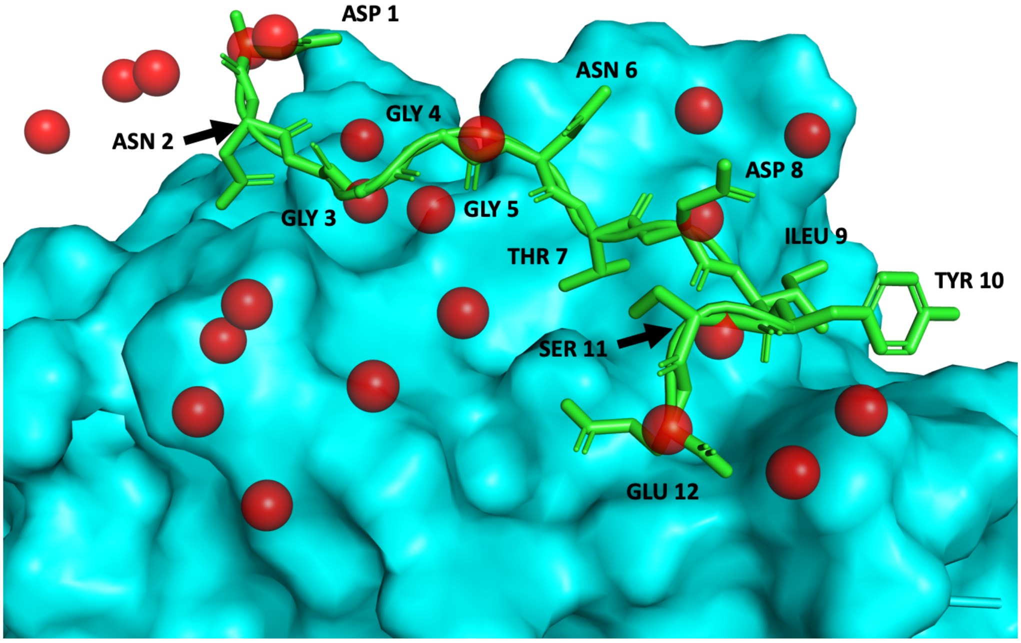

Figure S4. Structural modeling of PEP30-TWEAK complex. TWEAK and PEP30 shown in cyan (surface representation) and green (cartoon and stick representation) respectively. Several residues of PEP30 are shown for reference. Red spheres represent the anchor residues derived from the TWEAK-Antibody structure (PDB code: 4HT1[1]) used for peptide modeling (see Material and Methods and Supplementary file File2.coord.pdb).

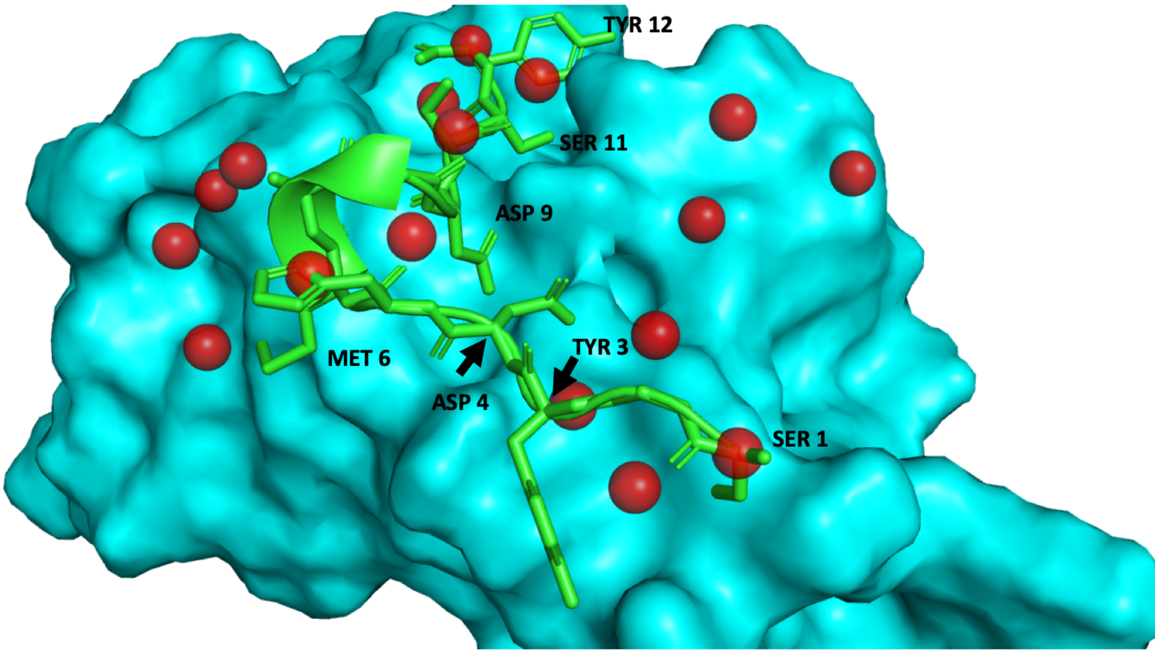

Figure S5. Structural model of PEP35-TWEAK complex. TWEAK and PEP35 shown in cyan (surface representation) and green (cartoon and stick representation) respectively. Several residues of PEP35 are shown for reference. Red spheres represent the anchor residues derived from the TWEAK-Antibody structure (PDB code: 4HT1[1]) used for peptide modeling (see Material and Methods and Supplementary file File3.coord.pdb).

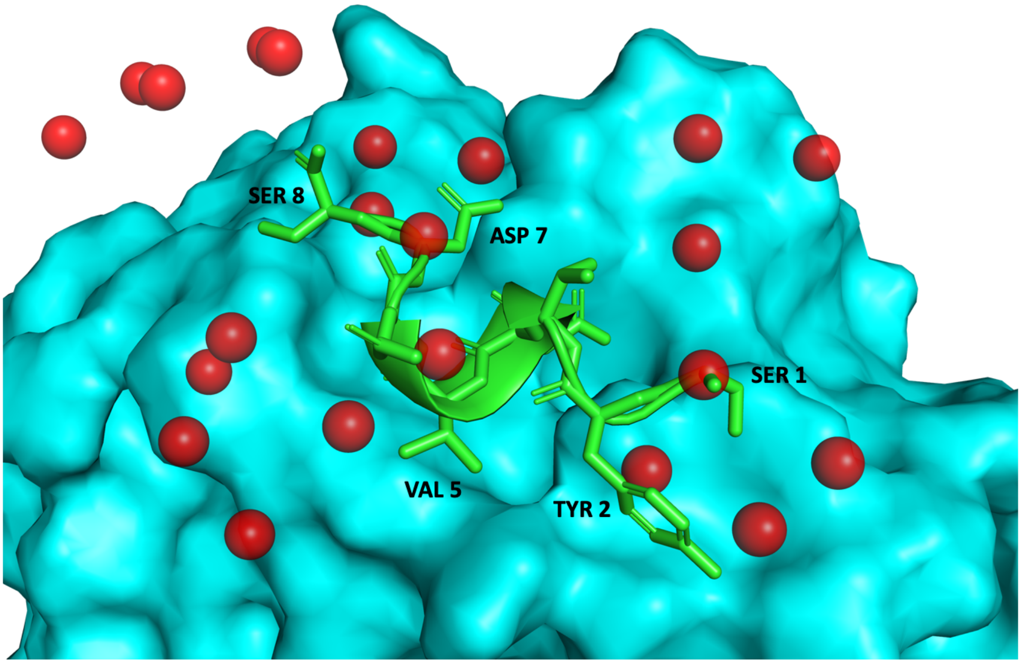

Figure S6. Structural model of PEP49-TWEAK complex. TWEAK and PEP49 shown in cyan (surface representation) and green (cartoon and stick representation) respectively. Several residues of PEP49 are shown for reference. Red spheres represent the anchor residues derived from the TWEAK-Antibody structure (PDB code: 4HT1[1]) used for peptide modeling (see Material and Methods and Supplementary file File4.coord.pdb).

Supplementary Tables.

Table S1. Analytical summary of designed and synthesized TWEAK-Fn14 inhibitory peptides.

ID	Pool[a]	Modeling ID	Peptide Sequence	Size	Charge (pH 1)	Charge (pH 7)	Analytical Characterization			
							MW (Da)[b]	RP-HPLC method[c]	t_R (min)	Purity (%)[d]
1	5	TWFA_10280	GNGTDDNSGTSY	12	1	-1	1186.11	0-25% B	5.68	92
2	3	TWFA_3299	YNVSVAGGHT	10	2	1,1	1003.08	5-40% B	5.08	99
3	1	TWNFA_1636	TKGGGTADA	9	2	1	775.82	0-10% B	3.45	97
4	1	TWNFA_1387	SGVSTAQLD	9	1	0	875.93	5-40% B	5.28	96
5	3	TWNFA_4288	LGAADQGLDI	10	1	-1	971.08	5-50% B	6.70	98
6	6	TWNFA_8523	GHALESSDLAGSAG	14	2	-0,9	1270.32	5-40% B	5.49	94
7	5	TWNFA_3718	HGTQSVSNFIGAH	13	3	1,2	1353.46	5-40% B	6.90	97
8	3	TWFA_31792	SSYGGYAEDA	10	1	1	1018.01	5-60% B	4.33	89
9	5	TWFA_15148	DLNYHGATAANY	12	2	0,1	1308.37	5-40% B	6.19	98
10	3	TWNFA_2203	SFHTLAGALG	10	2	1,1	972.11	5-60% B	6.43	92
11	1	TWNFA_5901	SGVAYQETT	9	1	0	954.01	5-60% B	5.87	92
12	4	TWFA_10298	GNLDDAIDHSY	11	2	-1,9	1218.25	5-60% B	5.40	93
13	1	TWFA_20354	YSGYFGGFY	9	1	1	1059.15	5-60% B	7.76	94
14	5	TWNFA_8477	AANSETASTSGTG	13	1	0	1152.14	0-20% B	5.30	89
15	3	TWFA_31907	SSFSYALSNT	10	1	1	1075.14	5-60% B	8.23	92
16	2	TWFA_16795	DDTSGALSS	9	1	-1	850.84	5-60% B	3.06	90
17	4	TWFA_3036	YNNVNLSDTGS	11	1	0	1182.21	5-60% B	4.38	92
18	3	TWFA_31985	SSMAYGFTNA	10	1	1	1047.16	10-50% B	5.30	99
19	2	TWNFA_1636	EDSLDTIAT	9	1	-2	963.01	5-60% B	5.45	91
20	3	TWNFA_1869	DLTADEDGLG	10	1	-3	1004.02	5-60% B	7.56	96
21	3	TWNFA_5451	GSGGDFHSLG	10	2	0,1	931.96	5-60% B	4.50	95
22	2	TWNFA_1636	DNGRENSWI	9	2	0	1089.13	5-50% B	5.98	99
23	3	TWNFA_1619	DIIMGVETAY	10	1	-1	1110.30	15-50% B	5.70	98
24	4	TWNFA_3137	DMDAELDSGA	10	1	-3	1022.06	5-60% B	4.79	95
25	2	TWNFA_1996	ISGGWDDTA	9	1	-1	919.95	5-40% B	6.28	97
26	4	TWNFA_1615	ESAVDGGVTA	10	1	-1	903.94	5-50% B	4.12	97
27	4	TWNFA_3119	VVVEDVNFDD	10	1	-3	1149.22	5-60% B	6.22	90
28	2	TWNFA_3092	GKDSGSHLG	9	3	1,1	855.91	5-60% B	2.00	94
29	4	TWNFA_3123	GDGTVSDTSS	10	1	-1	923.89	5-60% B	1.63	96
30	5	TWNFA_3655	DNGGGNTDIYSE	12	1	-2	1240.21	5-60% B	6.50	88
31	6	TWFA_21597	YDGGAATGDNFYDT	14	1	-2	1465.45	5-60% B	5.38	95
32	6	TWFA_30464	GSRDGGLITTSVSNY	15	2	1	1525.64	5-60% B	5.67	99
33	2	TWFA_33056	RYKGHIYNY	9	4	3,1	1212.38	5-60% B	6.82	94
34	2	TWFA_32757	RYYTAIGQY	9	2	2	1133.27	5-60% B	5.47	98
35	5	TWFA_31251	SWYDHMAGDSSY	12	2	-0,9	1417.48	5-60% B	5.93	95
36	4	TWFA_32845	RGHSGTDIGNY	11	3	1,1	1175.23	5-60% B	3.19	90
37	6	TWFA_5965	YNSGTGYAAQSDAR	14	2	1	1459.50	5-60% B	3.58	88
38	6	TWFA_33014	RLSIYYGASVGDSY	14	2	1	1559.70	5-60% B	6.46	84
39	6	TWFA_30152	LSGGFLGDGAAGQNDD	17	1	-2	1655.70	5-60% B	6.29	97
40	6	TWNFA_3713	LGHGFAGYAFAEERG	15	3	0,1	1580.72	5-60% B	6.30	97
41	5	TWNFA_3125	DSGGDFDVHHVA	12	3	-1,8	1254.28	5-60% B	4.36	90
42	1	TWFA_594	YNGHVKDD	8	2	0,1	945.99	20-40% B	2.10	90
43	5	TWNFA_3770	TTDGSTGDDLQA	12	1	-2	1179.16	5-60% B	3.43	96
44	4	TWFA_33135	RNSAYNDGHY	10	3	1,1	1195.22	5-60% B	3.27	92
45	1	TWFA_15353	DGGNY	5	1	0	523.50	5-60% B	1.50	90
46	1	TWNFA_2847	TGYAENV	7	1	0	751.79	5-60% B	3.65	91
47	4	TWNFA_1221	GSALDESTVV	10	1	-1	976.05	5-60% B	5.09	95
48	2	TWFA_7648	YNNDDAANP	9	1	-1	991.97	5-60% B	2.51	95
49	1	TWFA_23396	SYTDVQDS	8	1	-1	912.91	5-60% B	3.47	93
50	2	TWNFA_2782	GSAADTTRS	9	2	1	863.88	0-40% B	3.33	98

[a] Peptides were pooled according to size *i.e.,* pool 1 included peptides with 5 to 9 amino acid residues; pool 2 peptides with 9 residues; pool 3 with 10 residues length; pool 4 between 10 to 11; pool 5 between 12 to 13; and pool 6 peptide sizes between 14 to 17 amino acids. [b] Identity was established by HPLC-MS. [c] Analytical RP-HPLC was performed using linear gradients of solvent B (0.036% TFA in ACN) into A (0.045% TFA in H_2O) over 15 min at 1 mL/min flow rate and with UV detection at 220 nm. [d] Peptide purity was calculated using the percentage of peak area at the chromatographic profile.

Table S2. Normalized level of expression of Fn14 gene with and without TWEAK (25 ng/ml) in HT29, 435-P, 435-Br1 and BRV5 cell lines.

Cell Line	Technical Replicate	TWEAK treatment	Expression
HT29	1	NO	0.1839
HT29	2	NO	0.1754
HT29	3	NO	0.1792
HT29	1	YES	0.1964
HT29	2	YES	0.2154
HT29	3	YES	0.2052
435-P	1	NO	0.1885
435-P	2	NO	0.1743
435-P	3	NO	0.1553
435-P	1	YES	0.1395
435-P	2	YES	0.1292
435-P	3	YES	0.1408
435-Br1	1	NO	0.1705
435-Br1	2	NO	0.1786
435-Br1	3	NO	0.1755
435-Br1	1	YES	0.1600
435-Br1	2	YES	0.1676
435-Br1	3	YES	0.1719
BRV5	1	NO	0.0909
BRV5	2	NO	0.0826
BRV5	3	NO	0.0806
BRV5	1	YES	0.1340
BRV5	2	YES	0.1202
BRV5	3	YES	0.1441

Table S3. Normalized level of expression of genes in pool experiments using the housekeeping gene NARS2 as reference.

Gene	NT	T	Pool 1	Pool 2	Pool 3	Pool 4	Pool 5	Pool 6
**TRAF2**	0.9921	1.2452	-1.4615	0.0357	-0.0020	0.9037	-0.7081	-1.0050
**TNFRSF12A**	-0.5742	-0.4905	-1.2744	0.2624	0.5763	1.9755	-0.6786	0.2036
**MAP4K4**	-0.7183	-0.5276	-1.5660	0.3580	-0.1045	1.6294	-0.0491	0.9782
**NFKB2**	-0.8208	-0.0374	-1.3178	0.3794	-0.3141	2.0742	-0.1278	0.1645
**MAPK14**	0.1383	0.2377	-2.1390	0.4708	-0.1352	1.4430	-0.1234	0.1078
**JUN**	-1.0148	-1.0121	-0.9558	0.6237	-0.1854	1.4094	-0.1318	1.2670
**IL6**	-1.2090	-1.2337	-0.6000	0.5934	0.8668	1.2794	0.8111	-0.5080
**HSP90B1**	-0.5944	-0.5008	-1.7316	0.9650	-0.0707	1.4050	-0.1534	0.6809
**BIRC2**	-0.6423	1.2334	-1.6606	0.8368	-0.3445	1.0954	-0.5376	0.0195

Table S4. Normalized level of expression of genes in individual peptide experiments using the housekeeping gene NARS2 as reference.

Gene	NT	T	P45	P46	P42	P49	P3	P4	P11	P13	P1	P9	P30	P35	P41	P43	P7	P14
**TRAF2**	3.0439	2.2919	-0.5070	-0.1604	-0.1276	-0.5766	-0.4866	-0.2666	-0.4411	0.2550	-0.4104	-0.2477	-0.4386	-0.5479	-0.2014	-0.4089	-0.2173	-0.5527
**TNFRSF12A**	2.3399	1.8000	-0.0236	-0.2543	0.3098	-1.0819	1.1044	-0.6289	-0.2085	0.9100	-0.0875	0.0743	-1.1120	-1.0476	-0.6825	-1.1086	-0.5864	0.2834
**MAP4K4**	0.6666	0.6195	-0.0124	0.6365	1.1128	-1.3594	1.3444	0.6937	0.3403	1.6990	-0.3569	-0.1373	-1.5551	-1.7018	0.1763	-1.1253	-0.7317	-0.3092
**NFKB2**	-0.0595	1.4080	0.0982	1.3042	1.3869	-1.1526	1.2258	0.3908	0.6220	1.1240	-0.1191	-0.4681	-1.3232	-1.5063	-0.6577	-1.2088	-0.6607	-0.4038
**MAPK14**	2.1766	2.3319	0.1219	0.5199	0.3913	-0.5853	0.3511	-0.0192	0.3151	0.6701	-0.6461	-0.6137	-1.1124	-1.1107	-0.4551	-1.0059	-0.6198	-0.7097
**JUN**	-0.7833	-0.7938	-0.0963	-0.1078	-0.1905	-0.2062	0.5369	0.0589	0.0366	3.7769	-0.3055	-0.1385	-0.5095	-0.5085	-0.3008	-0.4883	0.2734	-0.2537
**IL6**	-1.4728	-1.5585	-0.2605	-0.0166	0.2346	-0.9717	1.1687	0.0669	-0.2862	2.8881	0.4124	-0.1350	-0.0450	0.1984	-0.7093	-0.5351	0.5279	0.4937
**HSP90B1**	0.7895	0.8798	0.0825	0.1322	0.9131	-0.4678	2.9702	0.3256	0.5337	-0.1010	-0.5389	-0.4890	-0.9208	-1.1014	-0.4648	-1.3086	-0.5127	-0.7215
**BIRC2**	0.9023	2.9803	-0.0012	-0.1180	-0.1362	-0.1150	1.1157	0.4721	0.5906	-0.8852	0.2639	-0.0012	-0.6095	-0.7468	-0.8982	-0.9963	-1.2320	-0.5850

Supplementary Files.

File1.coord.pdb. Atom coordinates in PDB (http://www.rcsb.org) format for TWEAK - peptide P43 complex.

File2.coord.pdb. Atom coordinates in PDB (http://www.rcsb.org) format for TWEAK - peptide P30 complex.

File3.coord.pdb. Atom coordinates in PDB (http://www.rcsb.org) format for TWEAK - peptide P35 complex.

File4.coord.pdb. Atom coordinates in PDB (http://www.rcsb.org) format for TWEAK - peptide P49 complex.

File5.xlsx. Normalized level of expression of probe genes.

References

[1] Lammens A, Baehner M, Kohnert U, Niewoehner J, von Proff L, Schraeml M, et al. Crystal structure of human TWEAK in complex with the Fab fragment of a neutralizing antibody reveals insights into receptor binding. PLoS One. 2013;8:e62697.
